# SF-MVPA: A from raw data to statistical results and surface space-based MVPA toolbox

**DOI:** 10.3389/fnins.2022.1046752

**Published:** 2022-11-21

**Authors:** Qiang Li, Dinghong Gong, Jie Shen, Chang Rao, Lei Ni, Hongyi Zhang

**Affiliations:** ^1^College of Education Science, Guizhou Education University, Guiyang, China; ^2^Guizhou Education University, Guiyang, China

**Keywords:** SF-MVPA, surface space-based MVPA, GUI, neural coding difference, fMRI

## Abstract

Compared with traditional volume space-based multivariate pattern analysis (MVPA), surface space-based MVPA has many advantages and has received increasing attention. However, surface space-based MVPA requires considerable programming and is therefore difficult for people without a programming foundation. To address this, we developed a MATLAB toolbox based on a graphical interactive interface (GUI) called surface space-based multivariate pattern analysis (SF-MVPA) in this manuscript. Unlike the traditional MVPA toolboxes, which often only include MVPA calculation processes after data preprocessing, SF-MVPA covers the complete pipeline of surface space-based MVPA, including raw data format conversion, surface reconstruction, functional magnetic resonance (fMRI) data preprocessing, comparative analysis, surface space-based MVPA, leave one-run out cross validation, and family-wise error correction. With SF-MVPA, users can complete the complete pipeline of surface space-based MVPA without programming. In addition, SF-MVPA is designed for parallel computing and hence has high computational efficiency. After introducing SF-MVPA, we analyzed a sample dataset of tonal working memory load. By comparison with another surface space-based MVPA toolbox named CoSMoMVPA, we found that the two toolboxes obtained consistent results. We hope that through this toolbox, users can more easily implement surface space-based MVPA.

## Highlights

- A surface space-based MVPA toolbox based on a graphical user interface and parallel computing design is proposed.
- SF-MVPA contains the complete pipeline of surface space-based MVPA, including raw data format conversion, surface reconstruction, raw fMRI data preprocessing, surface space-based MVPA, statistical analysis, leave one-run out cross validation, and family wise error correction.
- With SF-MVPA, users can perform surface space-based MVPA from raw fMRI data to statistical results without programming.

## Introduction

Multivariate pattern analysis (MVPA) is a booming neuroimaging data analysis technology (Mahmoudi et al., 2012; Kuntzelman et al., 2021). In contrast to traditional univariate statistical methods that perform calculations on a dependent variable, such as an Electroencephalography (EEG) channel or a voxel, MVPA takes into account the information encoded in the distributed neural activity patterns (Treder, 2020). This feature enables MVPA to detect neural coding differences. In the field of functional magnetic resonance (fMRI), MVPA has been successfully applied in many studies (Lee et al., 2011; Linke et al., 2011; Haxby et al., 2014; Jacobsen et al., 2015; Galeano Weber et al., 2017; Ogg et al., 2019; Czoschke et al., 2021; Mamashli et al., 2021; Putkinen et al., 2021). Most of these MVPA studies are based on volume space and use a 3D-spherical searchlight. This approach has several disadvantages (Oosterhof et al., 2011). First, due to the highly folded nature of the cerebral cortex, a spherical searchlight might include areas of the brain that are close in Euclidian space but not adjacent to each other. Second, a spherical searchlight might contain non-gray matter structures. Third, when located near the longitudinal fissure, a spherical searchlight might even collect information from both hemispheres. These defects might introduce interference into the local neural patterns.

To address these issues, researchers proposed surface space-based MVPA methods (Oosterhof et al., 2010; Chen et al., 2011; Li et al., 2022a,b). These MVPA methods obtain circular searchlights directly from the surface space (although there are differences in details of acquiring searchlights). This approach ensures that the obtained searchlights are from the adjacent brain regions, and the obtained local neural patterns are from the activity of the gray matter and the same hemisphere of the brain (Chen et al., 2011). Because of these advantages, compared with traditional volume space-based MVPA, surface space-based MVPA has better spatial specificity and is easier to visualize (Haynes, 2015). Although surface space-based MVPA has advantages over volume space-based MVPA, compared with the large number of toolboxes developed for volume space-based MVPA, few toolboxes have been developed for surface space-based MVPA.

Many excellent data analysis toolboxes have been proposed for volume space-based MVPA, including the MVPA-Light toolbox (Treder, 2020), MVPANI (Peng et al., 2020), Neurora (Lu and Ku, 2020), CoSMoMVPA (Oosterhof et al., 2016), PyMVPA (Hanke et al., 2009), Pattern Recognition for Neuroimaging Toolbox (PRoNTo) (Schrouff et al., 2013), the Neural Decoding Toolbox (NDT) (Meyers, 2013), The Decoding Toolbox (TDT) (Hebart et al., 2015), and the Deep-learning-based multivariate pattern analysis (dMVPA) (Kuntzelman et al., 2021). In contrast, few toolboxes have been developed for surface space-based MVPA. To our knowledge, CoSMoMVPA is the only toolbox that supports surface space-based MVPA. Although CoSMoMVPA is an excellent surface space-based toolbox, it only contains the pipeline after raw data preprocessing and is not based on a graphical interactive interface (GUI). Thus, users must program code when performing surface space-based MVPA, which is challenging for users without a programming background.

To make the implementation of surface space-based MVPA more convenient, we developed a GUI-based MALTAB toolbox named SF-MVPA. In contrast to traditional MVPA toolboxes, which often only contain calculation processes after data preprocessing (such as MVPA-Light and CoSMoMVPA), SF-MVPA contains the complete pipeline of surface space-based MVPA, including raw data format conversion, surface reconstruction, raw fMRI data preprocessing, surface space-based MVPA, statistical analysis, leave one-run out cross validation, and family wise error correction. Using this toolbox, by inputting parameters and clicking the mouse, the user can complete the complete analysis process of surface space-based MVPA with raw fMRI data.

Unlike volume space-based MVPA, the searchlights of surface space-based MVPA are surfaces, which makes the recognition of searchlights actually the recognition of images. In the field of image recognition, convolutional neural network (CNN) is a popular classifier and has been proven to have good image recognition ability. SF-MVPA uses CNN as an optional classifier and adopts the surface space-based MVPA algorithm of Li et al. (2022b). The algorithm is different from the other surface space-based MVPA algorithms in that it uses grid-like searchlights. Grid searchlights can be easily converted into 2D images, which makes the algorithm suitable for using CNN for image recognition. To be consistent with the traditional MVPA methods, SF-MVPA also includes support vector machine (SVM) as an optional classifier, which is one of the most commonly used machine learning algorithms in MVPA (Peng et al., 2020). To improve computational efficiency, SF-MVPA adopts the parallel computing design. Parallel computing design is used in processing processes such as data format conversion, surface reconstruction, data preprocessing, general linear model (GLM) analysis, and surface space-based MVPA.

After introducing SF-MVPA, we analyzed a sample dataset of tonal working memory load with SF-MVPA and CoSMoMVPA to make a comparison. We analyzed the neural coding differences between load 1 and load 4 (the number of tones held in mind) at the 7th and 11th seconds after stimulus onset. Although there were some differences, the two toolboxes obtained consistent results: at the 7th second, significant neural coding differences existed in the left supramarginal gyrus (SMG), precentral gyrus (PCG), supplement motor area (SMA), and bilateral superior temporal gyrus (STG). At the 11th second, significant neural coding differences existed in the bilateral PCG.

## Methods

### Requirements

**FIGURE 1:**
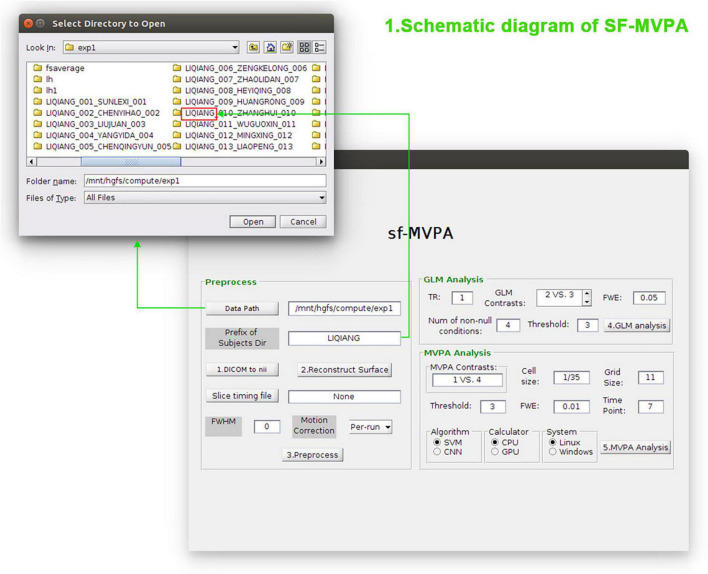
Schematic diagram of SF-MVPA. In the Preprocess module, the user needs to enter parameters such as the data path, prefix of subject folders, slice timing file, full width at half maxima (FWHM), and motion correction. After that, the user can click the buttons “1.DICOM to nii,” “2.Reconstruct Surface,” and “Preprocess” in sequence to convert the raw data format, reconstruct the surface, and preprocess the data. In the general linear model (GLM) analysis module, parameters such as TR, GLM contrasts, FWE threshold, number of non-null conditions, and vertex-wise threshold (Threshold) need to be entered before performing GLM analysis. In the multivariate pattern analysis (MVPA) module, parameters such as MVPA contrasts, cell size, grid size, vertex-wise threshold (Threshold), FWE threshold, time point (the time after stimulus onset), algorithm, and calculator need to be entered before performing surface space-based MVPA.

As shown in Figure 1, the calculation pipeline of SF-MVPA is divided into three parts: fMRI data preprocessing, contrast analysis (GLM), and surface space-based MVPA analysis (including statistical analysis and cluster permutation testing).

Functional magnetic resonance data preprocessing and contrast analysis need to call the functions of FreeSurfer^1^ and MATLAB.^2^ Hence, when performing fMRI data preprocessing and contrast analysis, users need to run SF-MVPA under a Linux operating system and need to install FreeSurfer and MATLAB. When executing surface space-based MVPA, SF-MVPA can run on a Linux or Windows operating system. This is because, on the one hand, the calculation of surface space-based MVPA does not need to call the functions of FreeSurfer; thus, SF-MVPA can run under a Windows operating system when performing surface space-based MVPA. On the other hand, some users use the Linux system in the virtual machine, and this situation may have poor compatibility with the graphics processing unit (GPU), which may interfere with the calculation based on the GPU. Because SF-MVPA is a parallel computing design, that is, each subject occupies a thread for computing, the hardware requirements are related to the amount of data. The recommended hardware requirements are that the number of CPU cores is greater than the number of subjects, and the available random access memory (RAM) is three times the raw functional data of subjects. However, SF-MVPA can still run on computers with lower than the recommended configuration (e.g., 8 cores and 32 GB RAM), but the computing speed will be reduced.

### Folder structure

**FIGURE 2:**
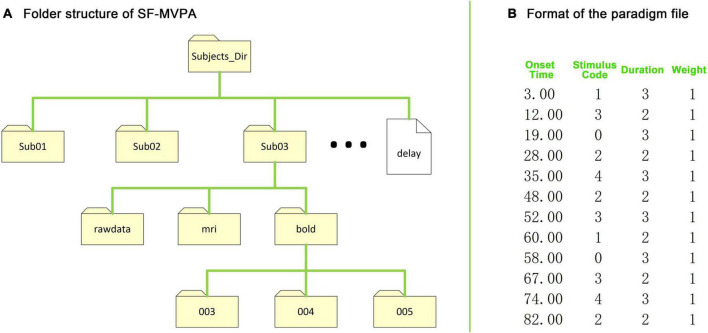
Folder structure of SF-MVPA and format of the stimulus paradigm file. **(A)** The folder structure of SF-MVPA. All data are stored in the folder “Subjects_Dir” (data path). Each subject has a folder (e.g., “Sub01”). Subject folders must have the same prefix (e.g., Sub). If the user wants to perform slice timing correction in the preprocessing process, then the user needs to copy the delay file that contains the slice timing information to “Subjects_Dir.” All raw data, including structural and functional data, need to be copied to the folder “rawdata.” After performing raw data conversion (by clicking “1.DICOM to nii”), the structural data are stored in the folder “mri” while the functional data are stored in run folders such as “003,” “004,” which are automatically created by SF-MVAP (the number corresponds to the run number). Before GLM analysis and multivariate pattern analysis (MVPA), the stimulus paradigm files need to be copied to the run folders. **(B)** The format of the stimulus paradigm files. The format has four columns, which are the onset times (seconds) of the stimuli, the stimulus codes (0 represent the baseline condition), the durations of stimuli (seconds), and the weights of stimuli.

SF-MVPA uses a simple folder structure. As shown in Figure 2A, each subject has a folder (i.e., “Sub01,” “Sub02,” etc., unlimited naming rules). Users need to create a folder named “rawdata” under each subject’s folder, and copy all the raw data, including structural data and functional data, into this folder (directly copy files, without folder). When performing raw data conversion, SF-MVPA will automatically create two folders named “mri” and “bold” and some subfolders under them under each subject folder. If users need to include slice timing correction in data preprocessing, they need to add a delay file in “Subjects_Dir.” The delay file has a single column of values, one for each slice. Each value is the slice delay measured as a fraction of the TR and ranges from +0.5 (beginning of the TR) to −0.5 (end of the TR). Before performing contrast analysis and surface space-based MVPA, the user needs to add paradigm files to run folders (i.e., “003,” “004,” etc.) of each subject. The format of the paradigm files is illustrated in Figure 2B.

### Data preprocessing

Data preprocessing of SF-MVPA includes data format conversion, surface reconstruction, and functional data preprocessing. These processes should be carried out in sequence. As illustrated in Figure 1, after specifying the path of “Subjects_Dir” and the prefix of the subject folders, the user can click the button “1.DICOM to nii” to realize data format conversion. In this step, the raw data of the structure image and function image is automatically detected and converted from digital imaging and communications in medicine (DICOM) format (Clunie, 2001) to NII format.^3^ The converted structural image data are saved in the “mri” folder, while the converted functional image data are saved in the “bold” folder. After data format conversion, the user can perform surface reconstruction by clicking the button “2.Reconstruct Surface.” The main surface reconstruction steps include motion correction and conform, non-uniform intensity normalization, skull stripping, volumetric labeling, white matter segmentation, smooth, inflate, spherical registration, surface extraction, cortical parcellation, etc. (Collins et al., 1994; Dale et al., 1999; Fischl et al., 1999a,b; Fischl and Dale, 2000). After surface reconstruction, the user can perform functional image data preprocessing by clicking the button “3.Preprocess.” Preprocessing stages include registration template creation, motion correction with motion parameters, slice-timing correction (if used), functional-anatomical registration, mask creation, global signal regression, resampling raw time series to standard spaces, and spatial smoothing (if used). In this step, there are several options to choose from. If the user selects “per-run,” motion correction and registration will use the middle time point of each run. If the user selects “per-session,” motion correction and registration will use the first time point of the first run. If the value of full width at half maxima (FWHM) is set to 0, spatial smoothing is not performed. If the value of the slice timing file is “None,” slice-timing correction is not performed. If the user needs to perform slice-timing correction, then the delay file needs to be specified.

### Contrast analysis

After data preprocessing, users can click the button “4.GLM analysis” to perform traditional contrast analysis. First-level and second-level fMRI data analyses were sequentially performed in this step. First-level analysis includes constructing the design matrix for each run, fitting the GLM, saving regression coefficients, and computing contrasts and significances of contrasts. After that, the analysis results of the first-level of all the sessions were concatenated into one multi-frame file, which is suitable for second-level analysis. A random-effects GLM was then conducted at the second-level analysis. To correct for multiple comparisons, the data were tested against the pre-cached simulation results of a Monte Carlo simulation. The user needs to specify the vertex-wise threshold [−log10(p)] and cluster-wise threshold (p) in SF-MVPA to correct for multiple comparisons. In addition, the TR, GLM Contrasts, and number of non-null conditions are also required for contrast analysis. GLM Contrasts allow formats such as (2 vs. 4), (1 3 vs. 2 4 5) and multiline input such as (1 vs. 2), another line, (3 vs. 4) to perform multiple comparative analyses at one time.

### Surface space-based multivariate pattern analysis

**FIGURE 3:**
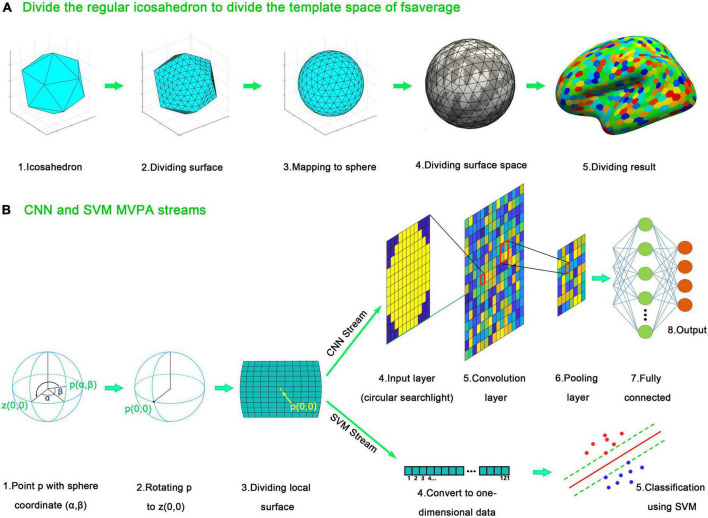
Surface space-based multivariate pattern analysis (MVPA) stream of SF-MVPA, which is adopted from Li et al. (2022b). **(A)** Surface dividing method. The spherical space of fsaverage is divided to avoid repeated calculation. The edges of the icosahedron are divided into 40 parts. The corresponding points are connected, forming new vertices. The vertices on the divided icosahedron are projected to a sphere and mapped to the spherical space of fsaverage. The vertices on the spherical space of fsaverage are grouped into the nearest vertices projected from the divided icosahedron, forming the downsampled spherical space of fsaverage. **(B)** Surface space-based MVPA streams. For each vertex *p* (α, β) on the downsampled spherical space of fsaverage, a rotation operation is performed. Vertex *p* is rotated to vertex *z* (0, 0). After that, a grid-like searchlight is acquired. The BOLD signals of the vertices (from the non-down-sampled fsaverage space) in the cells are averaged, forming an n1 × n1 image (n1 represents the number of cells of each side of the grid). A circle with a radius of n1/2 is applied to the image, forming a circular searchlight. The circular searchlight serves as the input data in the following analysis of the CNN stream. In the support vector machine (SVM) stream, the circular searchlight is converted into a one-dimensional vector and serves as the input data.

SF-MVPA adopts the surface space-based MVPA algorithm of Li et al. (2022b). The analysis stream is shown in Figure 3. The algorithm is based on the spherical template space of fsaverage, which is offered by FreeSurfer and has been included in SF-MVPA. This template space has a resolution of approximately 0.9 mm (Polimeni et al., 2019), which is much finer than the typical resolution of 2–3 mm of fMRI data (Lutti et al., 2013; Goense et al., 2016). Directly performing surface space-based MVPA in the template space of fsaverage cause repeated calculations and increase the calculation time. Therefore, the resolution of the template space is reduced. As illustrated in Figure 3A, the resolution reduction operation is based on the segmentation of the icosahedron. The edges of the icosahedron are divided into 40 equal parts. The corresponding points are connected to form vertices. These vertices are mapped onto a sphere and then transplanted to the spherical fsaverage template. Based on the distance, the vertices on the fsaverage template are grouped into these transplanted vertices. By doing this, the fsaverage template is divided into regular hexagons. These regular hexagons constitute new vertices, and the resolution of the fsaverage template is therefore reduced. By reducing the resolution, the number of vertices in the fsaverage template is reduced from 163,842 to 16,002, a difference of approximately 10 times. The fsaverage template has a resolution of approximately 0.9 × 0.9 × 0.9 mm ≈ 0.73 mm^3^. Reducing the resolution of the fsaverage template by a factor of 10 results in a resolution of 7.3 mm^3^ ≈ 1.9 × 1.9 × 1.9 mm, which is still better than or close to the resolution of typical fMRI images. Therefore, reducing the resolution of fsaverage does not affect the spatial accuracy of surface space-based MVPA but reduces the computational complexity.

SF-MVPA uses grid-like searchlights. As illustrated in Figure 3B, for each point *p* (α, β) on the fsaverage template (reduced resolution), a rotation operation is performed. The template is rotated so that point *p* is rotated to point *z* (0, 0). After that, a grid around point *p* (0, 0) is constructed. The user can set the parameters of the grid [size of the cell, number of cells of each side (n1)]. The default value is 1/35 radians per cell and 11 cells per side. BOLD signals within each cell were averaged and formed an n1 × n1 image. A circle with a radius of *r* (half the length of the grid side) was applied to the grid (data outside the circle were set to zero), forming a circular searchlight. After that, there are two surface space-based MVPA streams: the CNN stream and the SVM stream. In the CNN stream, a CNN composed of an input layer (n1 × n1), a convolution layer (n1 × n1, padding n1), a relu layer, a max pooling layer (2,2), a fully connected layer (equal to the number of categories to be classified), a softmax layer, and a classification layer is constructed. The CNN is trained by the training set and then used to classify the test set. A leave-one-run-out cross validation strategy is used. In this strategy, one run serves as the test set, and the remaining runs serve as the training set. Each run serves as the test set once. The classifying results of each run are averaged for each vertex of fsaverage (reduced resolution). After that, the averaged classifying results are compared with the probability of chance (1/the number of conditions involved) using a one-tailed *t*-test. The sizes of the activated clusters are detected with a recursive algorithm: if an activated vertex has not been grouped into a cluster, then it is grouped into a new cluster. If some of its adjacent vertices are also activated and have not been grouped into a cluster, then this vertex and these adjacent activated vertices are grouped into the same cluster. This grouping process is then recursively called by these activated adjacent vertices. The recursive process automatically stops when the entire cluster has been traversed. Then, this recursive process is executed by the remaining activated vertices that have not been grouped into a cluster. Using this recursive algorithm, all cluster sizes are obtained.

To correct for multiple comparisons, non-parameter permutation tests (Nichols and Holmes, 2002; Oosterhof et al., 2010; Czoschke et al., 2021; Erhart et al., 2021) were performed. The sign of the *t*-test result of each vertex is randomly flipped, and the size of the largest cluster is recorded. This process is repeated 2,000 times, simulating the largest size of the activated clusters generated by random data. The largest sizes of the 2,000 times are recorded and sorted in descending order. Then, the cluster sizes of the surface space-based MVPA are compared to the 20th simulated size, and only clusters larger than this simulated size are retained, corresponding to family-wise error (FWE) < 0.01 (20/2,000 = 0.01). Users can set the vertex-wise and family-wise thresholds in SF-MVPA.

In the SVM stream, the n1 × n1 image is transformed to a 1 × n1^2^ vector. This vector serves as the input data in the training and classifying processes. Except for this, the other steps, such as the *t*-test and the permutation tests, are the same as the CNN stream.

**FIGURE 4:**
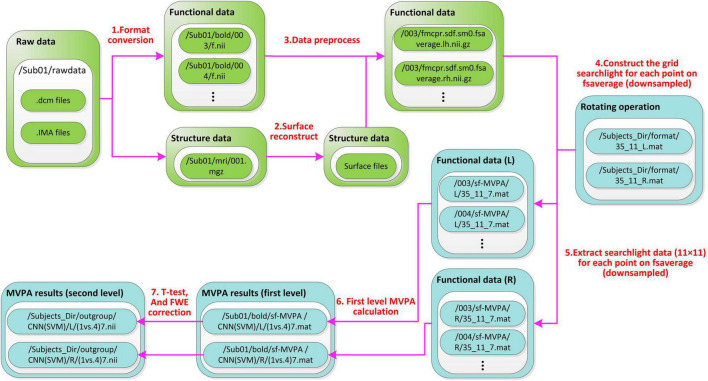
File stream of SF-MVPA. The preprocess module is green and the SF-MVPA module is cyan. Raw fMRI data (.dcm, IMA files, etc.) are stored in Sub0X/rawdata. After format conversion, structure data are stored in/Sub0X/mri/001.mgz and functional data are stored in/Sub0X/bold/00X/f.nii. Surface reconstruction produces a lot of files, which are stored in the folder/Sub0X/mri. After data preprocessing, functional data are stored in/Sub0X/bold/00X/fmcpr.sdf.sm0.fsaverage.l(r)h.nii.gz. The string “fmcpr.sdf.sm0.fsaverage.l(r)h.nii.gz” has meanings: “mc” means motion correction, “pr” means per-run, “sdf” means that the preprocessing process includes slice timing correction, “sm0” means do not perform spatial smoothing, “fsaverage” means that the data uses the template of fsaverage. The rotating operation produces two files (i.e., 35_11_L.mat and 35_11_R.mat), which are stored in the folder/Subjects_Dir/format. Both files contain a 16,002 × 124 cell array. Each row of the cell array corresponds to a point in the downsampled fsaverage template. The first column contains the spherical coordinates (α, β). The 2∼122 columns (11 × 11 = 121 cells) contain the vertices (of the undownsampled fsaverage template) covered by each cell. The 123th column contains the vertices (of the undownsampled fsaverage template) covered by the current point. The 124th column contains the adjacent points of the current point, which are used to determine the sizes of the clusters in the recursive process. Basing on the two cell arrays (lh and rh), the data of grid-searchlight are extracted from the preprocessed functional data. The data of grid-searchlight are cell arrays of 16,002 × 1, each cell contains an array of 33(34) × 121. The number 33(34) corresponds to the trials in a run. The file name (35_11_7.mat) of the grid-searchlight data means that: the cell size is 1/35 radian, each edge of the grid has 11 cells and the time point is the 7th second.

The file stream of SF-MVPA is shown in Figure 4.

### Analysis of the sample dataset

**FIGURE 5:**
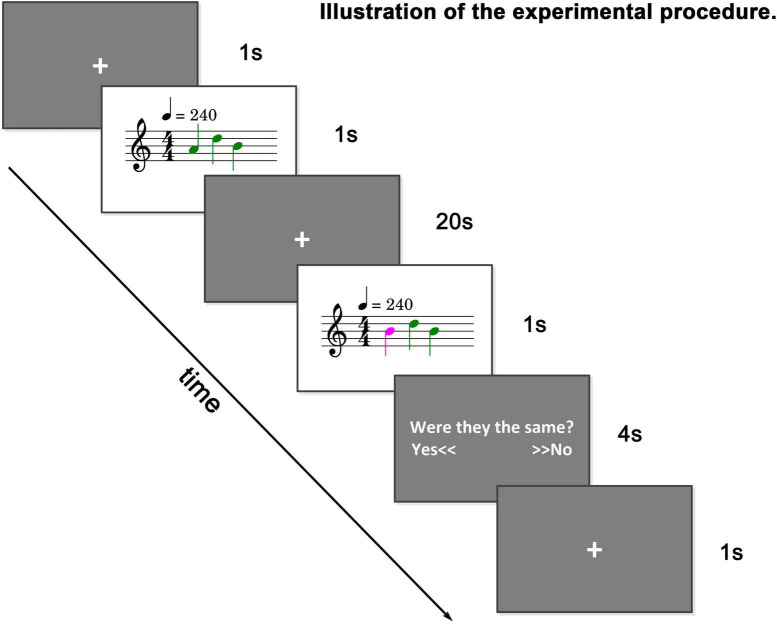
Illustration of sample dataset. A string of tones (0–4 tones) is played. After 20 s, another string of tones is played. The number of tones is equal. There is a 50% probability that one tone is different. Subjects were asked to judge whether the two strings of tones were the same.

An fMRI sample dataset is provided to familiarize users with and test the toolbox. The dataset is from a tonal working memory load experiment (Li et al., 2022a). In this experiment, the subjects were presented with a sequence of tones, and 20 s later, they were presented with another sequence of tones. The number of tones is equal, but there is a 50% probability that one tone is different (a difference of two natural tones). The subjects were asked to judge whether the two sequences of tones were the same with two buttons and were instructed to remember the first sequence of tones as clearly as possible to achieve the highest possible correct judgment rate. The experimental paradigm is illustrated in Figure 5.

The sequences of tones have 1–4 tones, corresponding to tonal working memory loads of 1–4. There was another condition in this experiment in which no sound was played. This condition was treated as load 0 and was used as a baseline condition. Each condition was repeated 20 times and presented in random order. There were 23 subjects (12 male, right handed, 18–23 years old) in total and the experiment was divided into three runs. The fMRI data were collected with a 3T Siemens Prisma_fit scanner. The T1-weighted structural data (1 × 1 × 1 mm) and T2*-weighted functional data (2.5 × 2.5 × 2.5 mm, TR = 1 s) were collected. For detailed scanning parameters, please refer to Li et al. (2022a).

For each subject, we copied all structural and functional raw images into the folder “rawdata.” After that, we copied the delay file, which contains the information of slice timing, into the folder “Subjects_Dir” (i.e., the parent folder of the subject folders, the folder where all the data were stored). Then, we specify the “Subjects_Dir” (data path), the prefix of subject folders (to enable SF-MVPA to know which folders are subject folders), the slice timing file (the delay file in “Subjects_Dir”), FWHM (0, which means do not perform spatial smoothing), and motion correction (per-run) in SF-MVPA. After doing this, we sequentially clicked “1.DICOM to nii,” “2.Reconstruct Surface,” and “3.Preprocess” to perform data preprocessing. It is worth mentioning that if a data processing process is executed, all buttons on the SF-MVPA are disabled, and the word “calculating” is displayed at the bottom of the SF-MVPA panel. After data preprocessing, we copied the stimulus paradigm files to the run folders (i.e., folders named like “003,” “004,” etc.). Then, we analyzed the local neural coding difference between load 1 and load 4 at the 7th and 11th seconds with SF-MVPA. We specified MVPA Contrasts “1 vs. 4” (allowing formats such as “2 3 vs. 1 4” and “0 1 2 3 4”), Cell size (1/35 radians), Grid size (11), vertex-wise threshold (3, corresponding to *p* < 0.001), FWE (0.01), and Time Point (7 or 11, one number in one time) in SF-MVPA. After that, we clicked the button “5.MVPA Analysis” to perform surface space-based MVPA and statistical analysis. In this step, both SVM and CNN were tested for comparison. To confirm whether SF-MVPA works properly, we performed the same analyses using CoSMoMVPA. Since CoSMoMVPA does not contain the functions of data preprocessing, we used the preprocessed fMRI data generated by SF-MVPA as the input data. CoSMoMVPA uses a geodesic distance metric method to construct disk searchlights (Oosterhof et al., 2010). In this manuscript, SF-MVPA used a radius of 100 (radius of the spherical space of fsaverage) × 1/35 × 11/2 ≈ 15.7. To be consistent with SF-MVPA, in the analysis of CoSMoMVPA, we used a radius of 15. In addition, CoSMoMVPA is also based on icosahedral segmentation. We used the same segmentation as SF-MVPA in the analysis of CoSMoMVPA, that is, each edge is divided into 40 equal parts. The analysis stream of CoSMoMVPA followed the instructions in the official website of CoSMoMVPA.^4^ The three-folded cross validation and FWE correction were the same as those for SF-MVPA.

## Results

**FIGURE 6:**
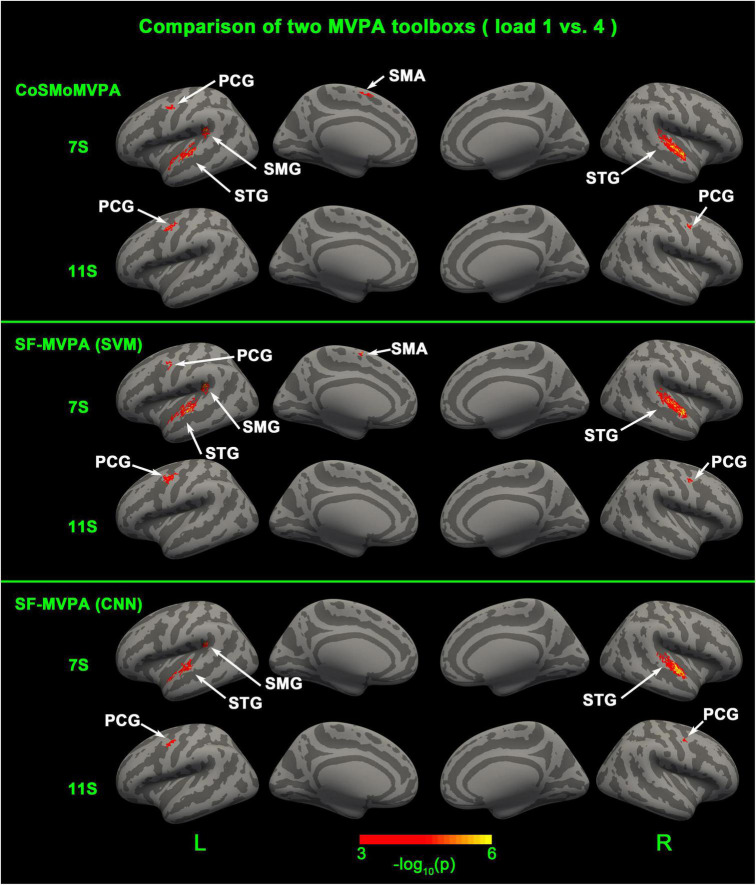
Surface space-based multivariate pattern analysis (MVPA) results (load 1 vs. load 4) of CosMoMVPA, SF-MVPA (SVM), and SF-MVPA (CNN).

As illustrated in Figure 6 and Table 1, CoSMoMVPA, SF-MVPA (SVM), and SF-MVPA (CNN) obtained similar results. For CoSMoMVPA, in the 7th second, neural coding differences between load 1 and load 4 existed in the bilateral STG and left SMG, PCG, and SMA. In the 11th second, neural coding differences between load 1 and load 4 existed in the bilateral PCG. For SF-MVPA (SVM), in the 7th second, neural coding differences between load 1 and load 4 existed in the bilateral STG, left PCG, SMG and SMA. In the 11th second, neural coding differences between load 1 and load 4 existed in the bilateral PCG. For SF-MVPA (CNN), in the 7th second, neural coding differences between load 1 and load 4 existed in the bilateral STG and left SMG. In the 11th second, neural coding differences between load 1 and load 4 existed in the bilateral PCG.

**TABLE 1:** Multivariate pattern analysis (MVPA) results of load 1 vs. load 4.

Toolbox	Time	Peak intensity	Size of cluster (mm^2)	Region	Talairach coordinates		
					
					*x*	*y*	*z*
CosMoMVPA	7 s	6.0	435	Superior temporal gyrus left	−59	−14	−2
	7 s	5.0	79	Superior temporal gyrus left	−47	−39	17
	7 s	4.9	147	Superior temporal gyrus left	−51	−2	−8
	7 s	6.5	103	Supramarginal gyrus left	−45	−39	23
	7 s	4.2	60	Supplement motor area left	−7	−1	56
	7 s	6.8	964	Superior temporal gyrus right	56	−16	3
	7 s	4.2	20	Superior temporal gyrus right	51	−33	15
	7 s	4.0	12	Superior temporal gyrus right	66	−25	5
	11 s	5.3	163	Precentral gyrus left	−47	3	40
	11 s	5.4	53	Precentral gyrus right	50	1	42
SF-MVPA (SVM)	7 s	6.8	1101	Superior temporal gyrus right	52	−11	−1
	7 s	7.0	633	Superior temporal gyrus left	−60	−16	2
	7 s	4.1	65	Precentral gyrus left	−46	3	40
	7 s	6.3	181	Supramarginal gyrus left	−46	−34	25
	7 s	5.5	26	Supplement motor area left	−11	9	60
	7 s	4.8	23	Superior temporal gyrus right	49	−33	15
	11 s	5.3	246	Precentral gyrus left	−45	0	42
	11 s	4.2	54	Precentral gyrus right	52	0	42
SF-MVPA (CNN)	7 s	5.5	346	Superior temporal gyrus left	−63	−17	0
	7 s	5.1	115	Superior temporal gyrus left	−53	2	−7
	7 s	5.4	92	Supramarginal gyrus left	−47	−36	26
	7 s	7.0	888	Superior temporal gyrus right	56	−8	0
	11 s	4.9	201	Precentral gyrus left	−49	−1	43
	11 s	4.0	34	Precentral gyrus right	51	−1	44

## Discussion

Surface space-based MVPA has attracted increasing attention because it can avoid the interference of non-gray matter tissues and avoid collecting information from discontinuous brain regions. Some surface space-based MVPA algorithms have been proposed (Oosterhof et al., 2010; Chen et al., 2011; Li et al., 2022a,b). However, due to the difficulty of obtaining searchlight from the surface space, these algorithms all use complex surface searchlight acquisition methods, which makes the programming implementation of these algorithms difficult. In addition, surface space-based MVPA often involves programming of multiple languages, such as shell and MATLAB (Li et al., 2022a), which makes it difficult for neuroimagers without a programming background to realize surface space-based MVPA. To address this, in this manuscript, we introduced a GUI and surface space-based MVPA toolbox named SF-MVPA. The aim of developing SF-MVPA is to reduce the programming effort of surface space-based MVPA and make surface space-based MVPA more accessible.

Theoretically, using SF-MVPA, users can realize surface space-based MVPA without programming, which is very friendly to users without programming background. A user of SF-MVPA only needs to place the raw fMRI data and paradigm files in the specified locations, and then can complete surface space-based MVPA step by step by inputting parameters and clicking the mouse. The complete pipeline of SF-MVPA includes raw data format conversion, surface reconstruction, raw fMRI data preprocessing, surface space-based MVPA, statistical analysis, leave one-run out cross validation, and family wise error correction. Without SF-MVPA, the programming implementation of these processes will be challenging and time-consuming.

In addition, due to its parallel computing design, SF-MVPA has a high computing efficiency. In the step of surface space-based MVPA, it took approximately 3 h for CoSMoMVPA to complete the calculation of a subject. Using SF-MVPA, due to the parallel computing design, the analysis of this step of all subjects was completed in approximately 3 h, which greatly shortened the calculation time. In addition to surface space-based MVPA, in SF-MVPA, other data processing steps such as data format conversion, surface reconstruction, data preprocessing, and GLM analysis also adopt a parallel computing design. When testing SF-MVPA, we found that a skilled user can complete the analysis of the sample dataset (from raw data to MVPA results) within 48 h.

In this manuscript, we offered a sample dataset and analyzed it with SF-MVPA and CoSMoMVPA. The results showed that these two toolboxes obtained similar results. SF-MVPA (SVM), SF-MVPA (CNN), and CoSMoMVPA found that in the 7th second, the bilateral STG, left SMG, and PCG had significantly different neural coding for load 1 and load 4, and in the 11th second, the bilateral PCG had significantly different neural coding for load 1 and load 4. The differences are: in the 7th second, CoSMoMVPA and SF-MVPA (SVM) found that the left SMA and PCG had significantly different neural coding for load 1 and load 4, while SF-MVPA (CNN) did not locate this area. Although there are differences, the area of the different areas is relatively small. Therefore, we argue that SF-MVPA and CoSMoMVPA have a similar ability to reveal neural coding differences. It is worth mentioning that SF-MVPA (CNN) does not achieve better results than SF-MVPA (SVM). In contrast, SF-MVPA (SVM) found activation of the left SMA and PCG at the 7th second, but SF-MVPA (CNN) did not. We think this may be caused by the CNN parameters not being set properly. In the process of developing SF-MVPA, we focused more on including the complete pipeline of surface space-based MVPA into SF-MVPA, and did not spend much time on the research of CNN parameters. We believe that with the version iteration of SF-MVPA in the future and the discovery of better and appropriate CNN parameters, the ability of SF-MVPA (CNN) to detect neural coding differences will be enhanced.

## Conclusion

SF-MVPA is an open source, GUI based MATLAB toolbox for surface space-based MVPA. It is easy to use and has high computing efficiency because of its parallel computing design. Unlike traditional MVPA toolboxes, which often only contain MVPA related calculations, SF-MVPA contains the complete pipeline of surface space-based MVPA, including raw data format conversion, surface reconstruction, data preprocessing, surface space-based MVPA, and statistical analysis. With SF-MVPA, the user can complete the from raw data to statistical results pipeline of surface space-based MVPA without programming, which reduces the difficulty of the implementation of surface space-based MVPA.

## Data availability statement

The original contributions presented in the study are publicly available. This data can be found here: https://pan.baidu.com/s/1kaMJmucuPGPD1UBhygvs5A, extracting code: liqi.

## Ethics statement

The studies involving human participants were reviewed and approved by the Ethics Committee of Southwest University. The patients/participants provided their written informed consent to participate in this study.

## Author contributions

QL designed the experiment, developed the toolbox, and wrote the manuscript. DG, JS, CR, and LN performed the experiment. HZ designed the experiment. All authors contributed to the article and approved the submitted version.

## Footnotes

1
https://www.freesurfer.net/

2
https://www.mathworks.com/products/matlab.html

3
https://nifti.nimh.nih.gov/

4
http://www.cosmomvpa.org/matlab/demo_surface_searchlight_lda.html

## Abbreviations

STG: superior temporal gyrus
SMG: supramarginal gyrus
PCG: precentral gyrus
SMA: supplementary motor area
IPL: inferior parietal lobe
EEG: electroencephalography
MVPA: multivariate pattern analysis
fMRI: functional magnetic resonance imaging
SVM: support vector machine
GLM: general linear model
BOLD: blood oxygen level dependent
CNN: convolutional neural network
GUI: graphical interactive interface
PRoNTo: pattern recognition for neuroimaging toolbox
NDT: the neural decoding toolbox
TDT: the decoding toolbox
dMVPA: the deep-learning-based multivariate pattern analysis
GPU: graphics processing unit
FWE: family-wise error.
